# Supplementary material for: Patterns of Intron Gain and Loss in Fungi
Source: PLoS Biol. 2004 Nov 30;2(12):e422. doi: 10.1371/journal.pbio.0020422 (PMC532390; doi:10.1371/journal.pbio.0020422)
Supplement: Table S1 — Also available at http://genes.mit.edu/NielsenEtAl/. (4.3 MB ZIP). [file pbio.0020422.st001.zip › NielsenEtAl/html/290.html]

AN3719.1.NCU02393.1.MG09565.1.FG06385.1


```
 CLUSTAL W (1.82) Multiple Sequence Alignments - Introns Inserted


Sequence 1: NCU02393.1	352 aa
Sequence 2: MG09565.1	356 aa
Sequence 3: FG06385.1	355 aa
Sequence 4: AN3719.1	361 aa
Alignment Length: 363 aa
Number Identitical Residues: 313 aa
Alignment Score (without introns) 12570


MG09565.1 	MSRANPPSNSSGSRKISFNVSEQYDIQDVVGEGAYGVVC2SAIHKPSGQKVAIKKITPFD
NCU02393.1	MSSAQ----RGGARKISFNVSEQYDIQDVVGEGAYGVVC2SAVHKPSGQKVAIKKITPFD
FG06385.1 	MSRANPP-NAAGSRKISFNVSEQYDIQDVVGEGAYGVVC2SAIHKPSGQKVAIKKITPFD
AN3719.1  	MVQQMPP--QGGSRKISFNVSDQYEIQDVIGEGAYGVVC2SAIHKPSGQKVAIKKITPFD
          	*    ..   .*:********:**:****:********* **:*****************

MG09565.1 	HSMFCLRTLREMKLLRYFNHENIISILDIQKPRSYETFNEVYLIQ0ELMETDMHRVIRTQ
NCU02393.1	HSMFCLRTLREMKLLRYFNHENIISILDIQKPRSYETFNEVYLIQ0ELMETDMHRVIRTQ
FG06385.1 	HSMFCLRTLREMKLLRYFNHENIISILDIQKPRSYESFQEVYLIQ0ELMETDMHRVIRTQ
AN3719.1  	HSMFCLRTLREMKLLRYFNHENIISILDIQRPRNYESFNEVYLIQ0ELMETDMHRVIRTQ
          	******************************:**.**:*:****** **************

MG09565.1 	DLSDDHCQYFIYQTLRALKAMHSANVLHRDLKPSNLLLNANCDLKVCDFGLARSAASQED
NCU02393.1	ELSDDHCQYFIYQTLRALKAMHSANVLHRDLKPSNLLLNANCDLKVCDFGLARSAASQED
FG06385.1 	DLSDDHCQYFIYQTLRALKAMHSANVLHRDLKPSNLLLNANCDLKVCDFGLARSAASQED
AN3719.1  	DLSDDHCQYFIYQTLRALKAMHSANVLHRDLKPSNLLLNANCDLKVCDFGLARSAASTDD
          	:******************************************************** :*

MG09565.1 	NSGFMTEYVATRWYRAPEIMLTFKEYTKAIDVWSVGCILAEMLSGKPLFPGKDY1HHQLT
NCU02393.1	NSGFMTEYVATRWYRAPEIMLTFKEYTKAIDVWSVGCILAEMLSGKPLFPGKDY1HHQLT
FG06385.1 	NSGFMTEYVATRWYRAPEIMLTFKEYTKAIDVWSVGCILAEMLSGKPLFPGKDY1HHQLT
AN3719.1  	NSGFMTEYVATRWYRAPEIMLTFKEYTKAIDVWSVGCILAEMLSGKPLFPGKDY1HHQLT
          	****************************************************** *****

MG09565.1 	LILDVLGTPTMEDYYGIKSRRAREYIRSLPFKKKVPFRTLFPKTSDLALDLLEKLLAFNP
NCU02393.1	LILDVLGTPTMEDYYGIKSRRAREYIRSLPFKKKVPFRTLFPNTSELALDLLEKLLAFNP
FG06385.1 	LILDVLGTPTMEDYYGIKSRRAREYIRSLPFKKKVPFRTLFPKTSDLALDLLEKLLAFNP
AN3719.1  	LILDVLGTPTMEDYYGIKSRRAREYIRSLPFKKKIPFKALFPKSNDLALDLLEKLLAFNP
          	**********************************:**::***::.:**************

MG09565.1 	VKRITVEEALKHPYLEPYHDPDDEPTAPPIPEEFFDFDKHKDNLSKEQLKQFIYQEIMR-
NCU02393.1	VKRITVEEALKHPYLEPYHDPDDEPTAPPIPEEFFDFDKHKDNLSKEQLKQLIYQEIMR-
FG06385.1 	VKRITVEEALKHPYLEPYHDPEDEPTAPPIPEEFFDFDKHKDNLSKEQLKQLIYQEIMR-
AN3719.1  	TKRITVEEALRHPYLEPYHDPDDEPTAPPIPEGFFDFDKNKDALSKEQLKSKYLAPIDSI
          	.*********:**********:********** ******:** *******.     *   

MG09565.1 	------
NCU02393.1	------
FG06385.1 	------
AN3719.1  	SYATIN
          	: :: .
```
